# First-dose and steady-state pharmacokinetics of orally administered crizotinib in children with solid tumors: a report on ADVL0912 from the Children’s Oncology Group Phase 1/Pilot Consortium

**DOI:** 10.1007/s00280-016-3220-6

**Published:** 2016-12-28

**Authors:** Frank M. Balis, Patrick A. Thompson, Yael P. Mosse, Susan M. Blaney, Charles G. Minard, Brenda J. Weigel, Elizabeth Fox

**Affiliations:** 1 The Children’s Hospital of Philadelphia, 3501 Civic Center Blvd, CTRB-4024, Philadelphia, PA 19104 USA; 2 University of North Carolina Lineberger Comprehensive Cancer Center, Chapel Hill, NC 27599 USA; 3 Baylor College of Medicine, Dan L Duncan Comprehensive Cancer Center, Houston, TX 77030 USA; 4 University of Minnesota, Masonic Cancer Center, Minneapolis, MN 55455 USA; 5 The Children’s Oncology Group Operations Center, Monrovia, CA 91016 USA

**Keywords:** Crizotinib, Pharmacokinetics, Childhood cancer, Children, ALK

## Abstract

**Purpose:**

Characterize the pharmacokinetics of oral crizotinib in children with cancer.

**Methods:**

Sixty-four children with solid tumors or anaplastic large-cell lymphoma (ALCL) enrolled on a phase 1/2 trial of the ALK, MET and ROS1 inhibitor, crizotinib, had pharmacokinetic sampling after the first dose (*n* = 15) or at steady state (*n* = 49). Dose levels studied were 100, 130, 165, 215, 280 and 365 mg/m^2^/dose administered twice daily. Two capsule and two oral liquid formulations were used over the course of the trial. Crizotinib was quantified with a validated HPLC/tandem mass spectrometry method with a lower limit of detection of 0.2 ng/mL. Pharmacokinetic parameters were derived using non-compartmental analysis.

**Results:**

Time to peak plasma concentration was 4 h. At 280 mg/m^2^ (MTD), mean (±SD) steady-state peak plasma concentration was 717 ± 201 ng/mL, and steady-state trough plasma concentration was 480 ± 176 ng/mL. At steady state, AUC_0–*τ*_ was proportional to dose over the dose range of 215–365 mg/m^2^/dose. Apparent clearance of crizotinib was 731 ± 241 mL/min/m^2^. Steady-state AUC_0–*τ*_ at 280 mg/m^2^/dose was 2.5-fold higher than the AUC_0–*∞*_ in adults receiving 250 mg (~140 mg/m^2^). Age, sex and drug formulation do not account for the inter-subject variability in AUC_0–*τ*_ at steady state. The accumulation index was 4.9, and the half-life estimated from the accumulation index was 36 h.

**Conclusions:**

The pharmacokinetics of oral crizotinib in children is similar to that in adults. Steady-state trough-free crizotinib concentrations in plasma at the MTD exceed inhibitory concentrations of crizotinib in ALCL cell lines.

**ClinicalTrials.gov identifier:**

NCT00939770.

## Introduction

Crizotinib is a small molecule inhibitor of multiple tyrosine kinases, including the anaplastic lymphoma kinase encoded by the *ALK* gene, hepatocyte growth factor receptor (c-met) encoded by *MET* and the tyrosine kinase, ROS encoded by *ROS1* [1]. Crizotinib is approved worldwide for the treatment of the subset of non-small-cell lung cancers (NSCLC) with rearrangements involving *ALK* [2–4]. Responses to crizotinib have also been reported in patients with anaplastic large-cell lymphoma (ALCL) and inflammatory myofibroblastic tumors [5, 6], both of which have a high incidence of *ALK* rearrangements, especially in children [7].

The adult recommended oral dose of crizotinib is 250 mg twice daily, which is equivalent to approximately 140 mg/m^2^/dose. The pharmacokinetics of crizotinib in adults has been studied after a single dose and at steady state (≥15 days) in patients with NSCLC using 250-mg formulated capsules and after a single 50-mg dose of an experimental intravenous formulation or a single 250-mg dose of three oral formulations [formulated capsules (FC), powder in capsules (PIC) and immediate-release tablets (IRT)] in healthy volunteers [8, 9]. The mean (C.V.) clearance (CL) of crizotinib was 47 (18%) L/h, the volume of distribution at steady state was 1770 (18%) L and the half-life (*T*_1/2_) was 39 (16%) h, following the intravenous dose. The absolute bioavailability (*F*) of the IRT formulation was 43%. After oral administration, the time to peak concentration (*T*_max_) was 5 h, the peak plasma concentration (*C*_max_) was 120–135 ng/mL, and the area under the plasma concentration–time curve extrapolated to infinity (AUC_0–*∞*_) was 2700–2900 ng h/mL. The three oral formulations were bioequivalent. The accumulation index (*R*) at steady state was 4.5.

A separate phase 1/2 trial of oral crizotinib administered twice daily using PIC, FC and two liquid formulations was conducted in children with solid tumors and ALCL [6]. Dose levels studied were 100, 130, 165, 215, 280 and 365 mg/m^2^/dose, and the maximum tolerated dose (MTD) was 280 mg/m^2^/dose, which is twofold higher than the adult recommended dose. Steady-state pharmacokinetic parameters in 18 children receiving 280 mg/m^2^/dose were briefly summarized in the previously reported phase 1/2 trial results [6]. This report describes in more detail the pharmacokinetics of crizotinib in children enrolled on the phase 1/2 trial and includes 64 subjects treated at the six dose levels and studied after the first dose (*n* = 15) or at steady state (*n* = 49). The previously reported 18 patients are included in this cohort.

## Materials and methods

### Drug

Crizotinib was provided by Pfizer Inc. (New York, NY) in four formulations over the course of the phase 1/2 clinical trial:

- PIC in 10, 50 and 100 mg strengths,
- FC (commercial formulation) in 150, 200 and 250 mg strengths,
- Powder in bottles (PIB) containing 2500 mg in a 4 oz vial that is reconstituted to a 25 mg/mL suspension,
- Oral solution (OS) containing 25 mg/mL in a sweetened and flavored aqueous vehicle. OS replaced PIB.

The OS is bioequivalent to FC in adults (Pfizer, data on file).

### Study design

The pediatric phase 1/2 trial of crizotinib (ADVL0912) was sponsored by Pfizer Inc. and conducted within the twenty-one member institutions of the Pediatric Phase 1 and Pilot Consortium plus five additional sites. The protocol was conducted in accordance with the Declaration of Helsinki and approved by the Institutional Review Boards at all participating institutions. Informed consent was obtained from all subjects who were 18 years or older or from the parents of subjects under the age of 18 years. The eligibility criteria, trial design and subject cohorts have been previously reported [6]. Crizotinib was administered orally, twice daily, continuously at doses of 100, 130, 165, 215, 280 and 365 mg/m^2^/dose on the phase 1 dose escalation portion of the trial and at a dose of 280 mg/m^2^/dose after this dose was identified as the MTD. For this report, the administered dose per m^2^ was calculated by dividing the patient’s dose (in mg) that was determined by a dosing nomogram in the protocol by their body surface area. In subjects receiving capsules, the administered dose per m^2^ often deviated slightly from the dose level because of dosing limitations imposed by capsule size.

### Sample collection and processing

Subjects studied after their first dose of crizotinib had 2 mL blood samples collected into potassium EDTA-containing tubes prior to the dose and 0.5, 1, 2, 4, 6, 8 and 24 h after the morning dose. The evening dose of crizotinib was not administered on day 1 in these subjects. A trough blood sample was also drawn prior to the morning dose on day 7. Subjects studied at steady state (day 15–28 of cycle 1) had blood samples drawn prior to their first dose on day 1, prior to the morning dose at steady state and then 1, 2, 4, 6–8 h after the morning dose. Blood samples were protected from light and placed on ice. Plasma was separated by centrifugation at 1700*g* for 10 min at 4 °C and immediately frozen at −20 to −70 °C until assayed.

### Crizotinib assay

Plasma concentrations of crizotinib were quantified by Covance Bioanalytical Services (Indianapolis, IN) using a previously described, validated high-pressure liquid chromatography (HPLC), tandem mass spectroscopic method with a lower limit of quantification of 0.2 ng/mL [8].

### Pharmacokinetic analysis

Crizotinib plasma concentration–time data were analyzed using model-independent methods. The area under the crizotinib plasma concentration–time curve to the last measured time point (AUC_0–tlast_) was derived using the linear trapezoidal rule. The limited sampling duration did not allow for accurate estimation of the terminal slope of the plasma concentration–time curve after the first dose. As a result, the terminal *T*_1/2_ could not derived, and the AUC could not be accurately extrapolated to infinity (AUC_0–*∞*_) in the subjects studied after the first dose. For calculating the steady-state AUC over the 12-h dosing interval (*τ*), which is equivalent to AUC_0–*∞*_ after a single dose, the trough concentration drawn prior to the dose was also used as the 12-h post-dose concentration. The average plasma crizotinib concentration at steady state ($C_{\text{ave}}^{\text{ss}}$) was derived by dividing the AUC_0–*τ*_ by *τ* (12 h), and the apparent clearance (CL/F) of crizotinib is the dose divided by AUC_0–*τ*_.

*R* was derived by dividing the day 7 steady-state trough concentration ($C_{{12{\text{h}}}}^{\text{ss}}$) by the concentration 12 h after the first dose (C_12h_). C_12h_ was estimated by extrapolation using the slope estimated from the Ln transformed 8 and 24 h plasma concentrations. *T*_1/2_ was estimated from the mean *R* by rearranging the equation for calculating *R* from the *T*_1/2_:

$$ R = \frac{1}{{\left[ {1 - {\text{e}}^{{\left( { - \frac{0.693}{{t_{1/2} }} \cdot \tau } \right)}} } \right]}}.$$

### Statistical analysis

A linear multiple regression model was used to assess the relationship between AUC_0–*τ*_ normalized to the administered dose/m^2^ at steady state and age, gender and drug formulation using the *lm* function in R statistical software package (The R Foundation for Statistical Computing, http://www.R-project.org).

## Results

### Subjects

Seventy-five subjects had pharmacokinetic blood samples drawn after their first dose of crizotinib (*n* = 15) or at steady state immediately prior to and after their morning dose (*n* = 60). The data from 11 subjects studied at steady state were not evaluable because the trough sample was not drawn at the appropriate time (*n* = 6), samples were missed (*n* = 2), the dose of crizotinib had been reduced for prior toxicity (*n* = 1), the dose was spit out (*n* = 1), or the preceding evening dose had not been given (*n* = 1). The characteristics of the 64 subjects with evaluable data are listed in Table 1.

**Table 1:** Subject characteristics

	First dose	Steady state
Number	15	49
Median (range) age (year)	11.9 (5.7–21.4)	10.1 (2.6–22)
Male/female	10:5	23:26
Dose level (mg/m^2^/dose)		
100	3	
130	3	
165	7	1
215	2	5
280		36
365		7
Formulation		
Powder in capsule	14	28^a^
Powder in bottle	1	8
Formulated capsule		9
Oral solution		4

^a^One subject received a combination of powder in capsule and powder in bottle

### First-dose pharmacokinetics

**Fig. 1:**
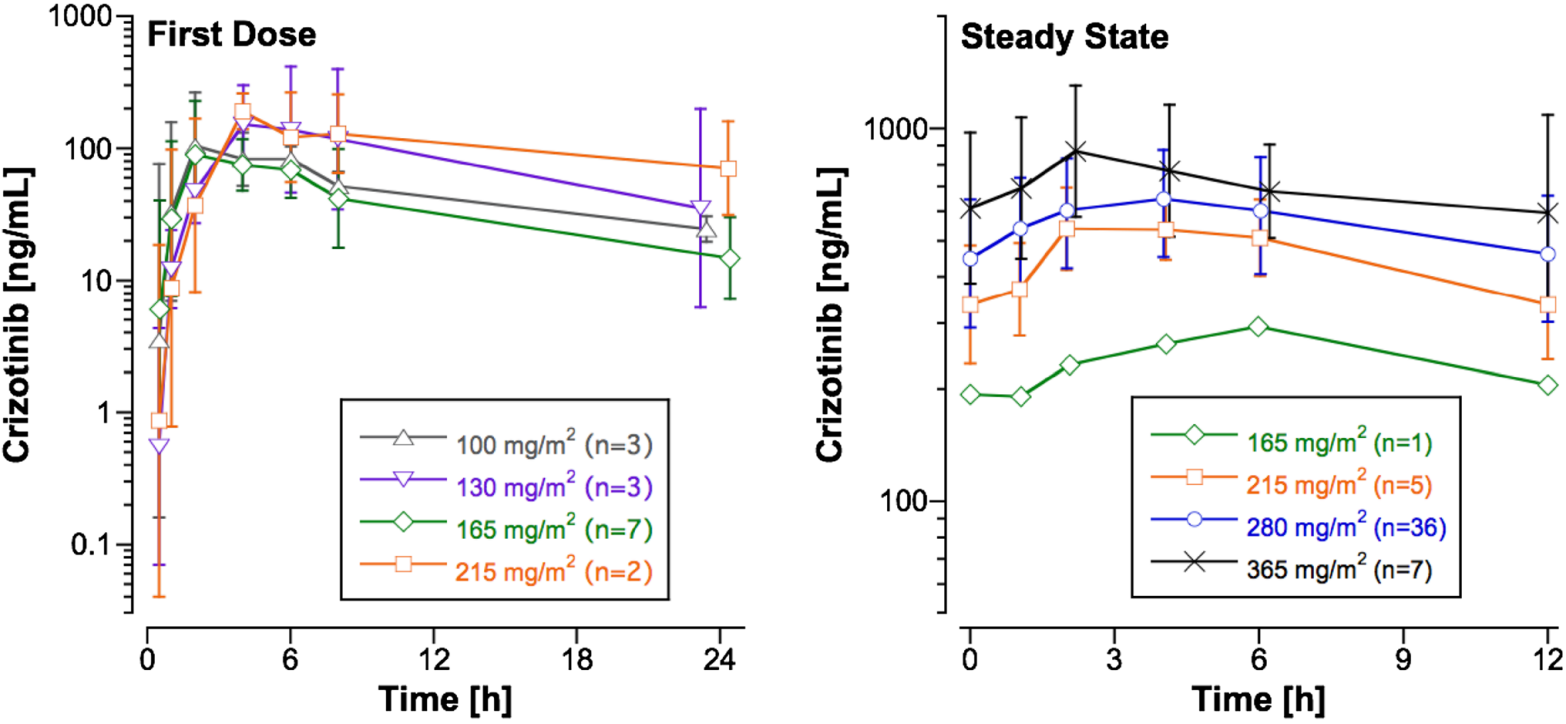
Plasma concentration–time profiles in subjects studied after the first dose and at steady state at 6 dose levels. *Points* are the geometric mean and *error bars* are the SD

**Fig. 2:**
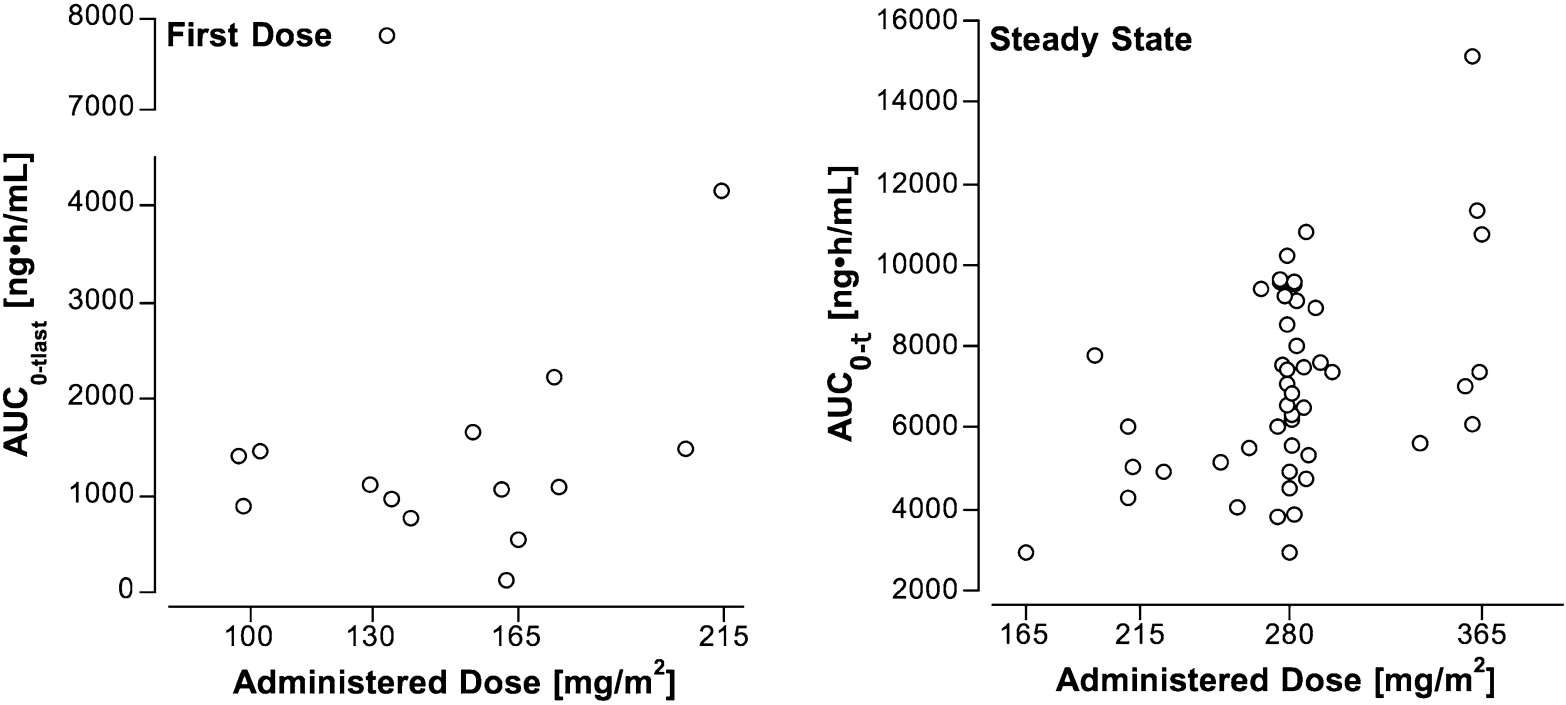
Relationship between AUC to the last measured time point (24 h) for subjects studied after the first dose or AUC over the 12-h dosing interval at steady state and the administered dose of oral crizotinib normalized to subject’s body surface area

Figure 1 shows the mean plasma crizotinib concentration–time profiles for subjects studied after the first dose at four dose levels, and Table 2 provides the pharmacokinetic parameters. Figure 2 shows the relationship of dose to AUC_0–tlast_. AUC_0–tlast_ does not appear to increase in proportion to the dose over the twofold dose range (100–215 mg/m^2^). This may reflect the substantial inter-subject variability (C.V. for the AUC_0–tlast_ normalized to the administered dose per m^2^ was 105%), as well as the small cohort size and the influence of an outlier at 130 mg/m^2^/dose (Fig. 2). The mean *R* across the four dose levels was 4.9, and the *T*_1/2_ of crizotinib estimated from the mean *R* was 36 h.

**Table 2:** Pharmacokinetic parameters for oral crizotinib studied after the first dose in children with solid tumors or ALCL at four dose levels ranging from 100 to 215 mg/m^2^/dose

Dose level (mg/m^2^/dose)	Number of subjects	*C*_max_ (ng/mL)	*T*_max_ (h)	AUC_0–tlast_ (ng h/mL)	*R*
100	3	144 ± 97	3.3 ± 2.2	1260 ± 320	2.8 ± 1.6
130	3	236 ± 217	4.7 ± 1.1	3300 ± 3913	5.8 ± 2.9
165	7	145 ± 70	3.7 ± 2.4	1208 ± 558	5.2 ± 2.1
215	2	196 ± 60	4.0 ± 0	2820 ± 1890	5.0 ± 2.3

Fourteen of the 15 subjects received the PIC formulation. Values represent the mean ± SD

### Steady-state pharmacokinetics

The crizotinib plasma concentration–time profile over the 12-h dosing interval at steady state for the dose levels ranging from 165 to 365 mg/m^2^ is shown in Fig. 1, and the mean (±SD) steady-state pharmacokinetic parameters are listed in Table 3. At steady state, the increase in AUC_0–*τ*_ is dose-proportional over the narrow dose range (215 to 365 mg/m^2^). The variability appears to be lower at steady state (C.V. for the AUC_0–*τ*_ normalized to the administered dose per m^2^ was 30%) than after the first dose. The mean (±SD) CL/F of crizotinib after oral dosing in children was 731 ± 241 mL/min/m^2^. The ratio of the steady-state peak plasma concentration ($C_{ \hbox{max} }^{\text{ss}}$) to $C_{{12{\text{h}}}}^{\text{ss}}$ at the 280 mg/m^2^ dose level (*n* = 36) was 1.6 ± 0.5, which reflects the intra-subject variation in plasma crizotinib concentrations over the dosing interval at steady state.

**Table 3:** Pharmacokinetic parameters for oral crizotinib studied at steady state in children with solid tumors or ALCL at four dose levels ranging from 165 to 365 mg/m^2^/dose

Dose level (mg/m^2^/dose)	Number of subjects	*C*_max_ (ng/mL)	*T*_max_ (h)	AUC_0–*τ*_ (ng h/mL)	$C_{\text{ave}}^{\text{ss}}$ (ng/mL)	CL/F (mL/min/m^2^)	$C_{{12{\text{h}}}}^{\text{ss}}$ (ng/mL)
165	1	294	6	2950	246	735	205
215	5	601 ± 118	4.0 ± 1.4	5630 ± 1370	469 ± 114	652 ± 159	354 ± 133
280	36	717 ± 201	3.8 ± 1.7	6990 ± 2080	582 ± 173	736 ± 255	480 ± 176
365	7	972 ± 210	2.1 ± 0.1	8770 ± 2740	731 ± 228	731 ± 223	650 ± 330

Values represent the mean ± SD

**Fig. 3:**
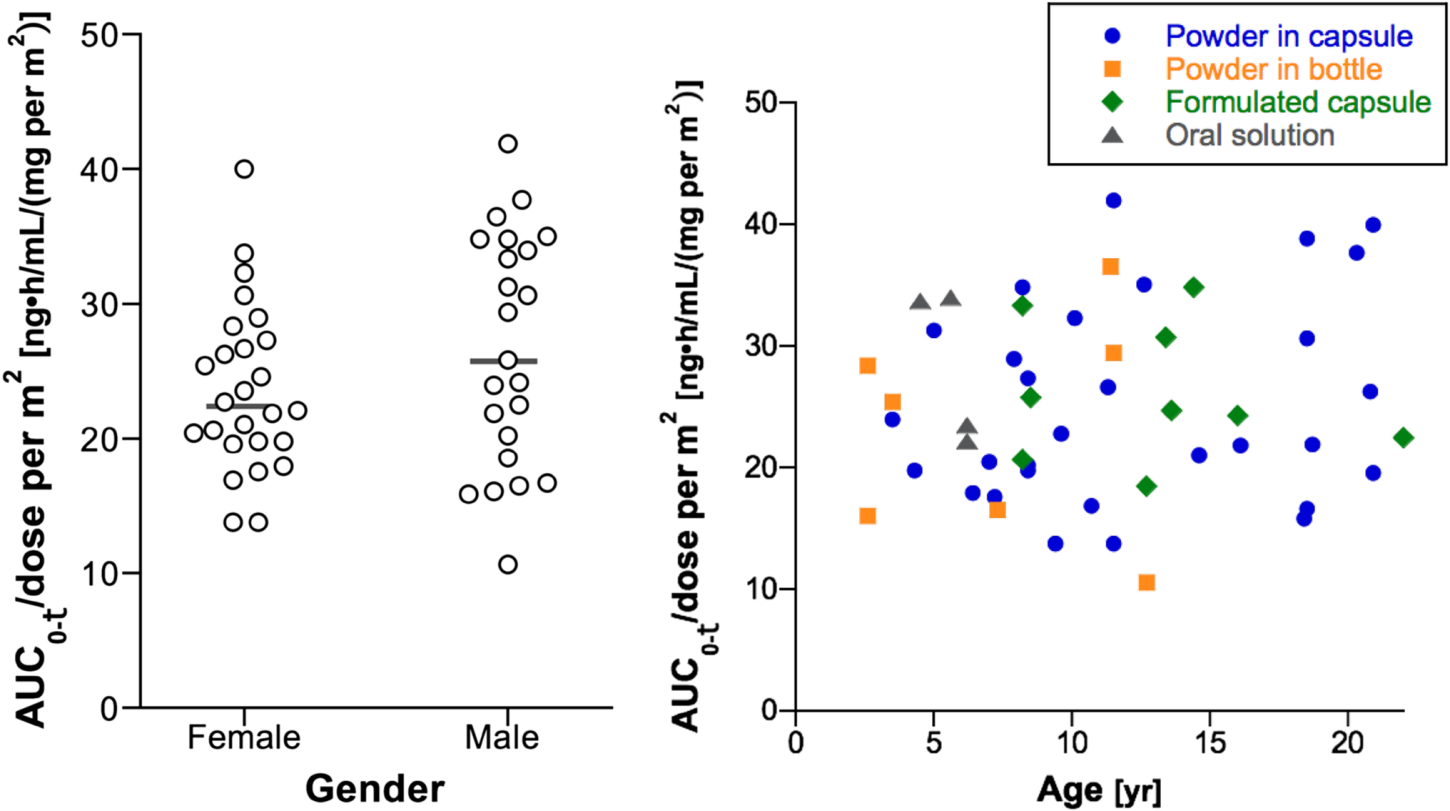
Relationship of gender (*left*) and age by crizotinib formulation (*right*) to the AUC_0–*τ*_ at steady state normalized to the administered dose per m^2^

A multiple regression analysis evaluating the impact of age, gender and drug formulation on the AUC_0–*τ*_ normalized to the administered dose per m^2^ at steady state in 49 subjects demonstrated that these characteristics did not account for the variability in AUC_0–*τ*_ (multiple *R* ^2^ = 0.11; *p* = 0.40). Figure 3 shows the relationship between the normalized AUC_0–*τ*_ and age, gender and drug formulation.

## Discussion

We characterized the pharmacokinetics of orally administered crizotinib in children after the first dose and at steady state (≥14 days of continuous twice daily dosing), at 6 dose levels ranging from 100 to 365 mg/m^2^/dose using four drug formulations. Crizotinib was dosed based on body surface area using a dosing nomogram. Despite differences in study design and sampling times, the pharmacokinetics of crizotinib in children appears to be similar to adults. The CL/F of crizotinib in adults was 850 mL/min/m^2^ after a single fixed 250-mg dose in healthy volunteers and 600 mL/min/m^2^ at steady state in adults with NSCLC [8, 9]. These values are comparable to the mean CL/F at steady state in children of 730 mL/min/m^2^. The $C_{ \hbox{max} }^{\text{ss}}$ in adults with NSCLC and children is also comparable when adjusted for differences in the dose.

Blood sampling after a single oral 250-mg dose of crizotinib in healthy volunteers extended to 144 h, which allows for an accurate determination of the 35 h terminal *T*_1/2_ [8]. It is not feasible to withhold crizotinib dosing for 6 d to allow for extended blood sampling in children with cancer, and estimating the *T*_1/2_ from the samples collected over 24 h yields a mean *T*_1/2_ of 11 h, which is an underestimation of the true *T*_1/2_ rather than a true difference in the disposition of crizotinib in children versus adults. The *R* calculated from the C_12h_ after the first dose and the day 7 trough concentration in children was 4.9, which is similar to 4.5 in adult [9], and the *T*_1/2_ estimated from the mean *R* in children was 36 h, which is similar to the *T*_1/2_ in adult healthy volunteers. Although this is an indirect method of estimating the *T*_1/2_, it is likely to be more accurate than estimating it directly from the concentration–time curve after the first dose because of the limited sampling duration.

At the MTD (280 mg/m^2^/dose) of crizotinib in children, the mean (±SD) $C_{\text{ave}}^{\text{ss}}$ is 580 (±170) ng/mL, and the mean $C_{{12{\text{h}}}}^{\text{ss}}$ is 480 (±180) ng/mL. Plasma protein binding of crizotinib in adults is 90% [9], and free $C_{\text{ave}}^{\text{ss}}$ and $C_{{12{\text{h}}}}^{\text{ss}}$ are estimated to be 60 and 50 ng/mL, respectively. These free drug concentrations exceed the 10 ng/mL IC_50_ of crizotinib in ALCL cell lines, Karpas299 and SU-DHL-1 that express the NPM-ALK fusion protein [10], consistent with the high objective response rate to crizotinib in children with ALCL treated on the phase 1/2 trial even at doses below the MTD [6].

The lactam metabolite of crizotinib, PF-06260182, is measurable in plasma, but at substantially lower concentrations than the parent drug [8]. Although PF-06260182 is active, it is considerably less potent than crizotinib, and it is not thought to contribute significantly to the in vivo activity of crizotinib [9, 11]. For this reason, we did not monitor PF-06260182 plasma concentrations in children.

In summary, the pharmacokinetics of oral crizotinib in children is similar to that in adults. A more palatable formulation is currently being evaluated in children. Clinical trials of crizotinib alone and in combination with chemotherapy in children with neuroblastoma, other solid tumors and ALCL are ongoing.

## Abbreviations

ALCL: Anaplastic large-cell lymphoma
AUC: Area under the plasma concentration–time curve
*C*_12h_: Plasma concentration 12-h post-first dose (trough)
$C_{{12{\text{h}}}}^{\text{ss}}$: Plasma concentration 12-h post-dose (trough) at steady state
$C_{\text{ave}}^{\text{ss}}$: Average plasma concentration at steady state
CL: Clearance
CL/F: Apparent clearance
*C*_max_: Peak plasma concentration
$C_{ \hbox{max} }^{\text{ss}}$: Peak plasma concentration at steady state
*F*: Fraction of the dose that is absorbed (bioavailable)
FC: Formulated capsule
HPLC: High-pressure liquid chromatography
IRT: Immediate-release tablet
MTD: Maximum tolerated dose
NSCLC: Non-small-cell lung cancer
OS: Oral solution
PIB: Powder in bottle
PIC: Powder in capsule
*R*: Accumulation index
*τ*: Dosing interval
*T*_1/2_: Half-life
*t*_last_: Time of last measured plasma concentration
*T*_max_: Time post-dose that the *C*_max_ is reached
